# Circular RNA profiles in mouse lung tissue induced by radon

**DOI:** 10.1186/s12199-017-0627-6

**Published:** 2017-04-07

**Authors:** Weiwei Pei, Lijing Tao, Leshuai W. Zhang, Shuyu Zhang, Jianping Cao, Yang Jiao, Jian Tong, Jihua Nie

**Affiliations:** 1 School of Public Health, Medical College of Soochow University, Suzhou, 215123 China; 2 Jiangsu Key Laboratory of Preventive and Translational Medicine for Genetic Diseases, Suzhou, 215123 China; 3 School of Radiation Medicine and Protection and Jiangsu Provincial Key Laboratory of Radiation Medicine and Protection, Medical College of Soochow University, Suzhou, 215123 China; 4 Collaborative Innovation Center of Radiation Medicine of Jiangsu Higher Education Institutions and School for Radiological and Interdisciplinary Sciences (RAD-X), Soochow University, Suzhou, 215123 China

**Keywords:** Radon, Circular RNA, Damage, Mouse, Lung tissue

## Abstract

**Background:**

Radon is a known human lung carcinogen, whose underlying carcinogenic mechanism remains unclear. Recently, circular RNA (circRNA), a class of endogenous non-protein coding RNAs that contain a circular loop, was found to exhibit multiple biological effects. In this study, circRNA profiles in mouse lung tissues between control and radon exposure were analyzed.

**Methods:**

Six mice were exposed to radon at concentration of 100,000 Bq/m^3^, 12 h/d, for up to cumulative doses of 60 working level months (WLM). H&E staining and immunohistochemistry of caspase-3 were used to detect the damages in lung tissue. The lung tissue of control and exposed group were selected for circRNA microarray study. The circRNA/microRNA interaction was analyzed by starBase prediction software. 5 highest expressing circRNAs were selected by real-time PCR to validate the consistency in mouse lung tissue exposed to radon.

**Results:**

Inflammatory reaction was found in mouse lung tissue exposed to radon, and caspase-3 expression was significantly increased. Microarray screening revealed 107 up-regulated and 83 down-regulated circRNAs, among which top 30 circRNAs with the highest fold changes were chosen for further analysis, with 5 microRNAs binding sites listed for each circRNA. Consistency of the top 5 circRNAs with the highest expressions were confirmed in mice exposed with 60WLM of radon.

**Conclusion:**

Mouse lung tissue was severely injured when exposed to radon through pathological diagnosis and immunohistochemical analysis. A series of differentially expressed circRNAs demonstrated that they may play an important role in pulmonary toxicity induced by radon.

## Background

Radon is the most notorious environmental radioactive gas and has been categorized by the World Health Organization as a carcinogenic substance that causes lung cancer. In the United States, an estimated 21,000 lung cancer deaths annually are attributed to radon [1–4]. Radon decays with the emission of α particles and has a half-life of 3.8 days. When radon is inhaled, α particles could affect lung tissue and may cause lung cancer via DNA and oxidative damages [5, 6]. However the causative effect of radon on lung cancer and its specific mechanisms remain unclear, and one of the carcinogenic mechanisms is thought to be DNA damage through reactive oxygen species produced by α particles [7]. Loss of heterozygosity analysis of the Cdkn2a locus, including the Ink4a and Arf genes, was performed on 33 radon-induced rat lung tumors, and DNA losses were observed in 50% of cases [8]. On the other hand, apoptosis, also known as programmed cell death, can be activated through the intrinsic mitochondrial-dependent pathway, by releasing cytochrome C which binds apoptotic protease activating factor-1 and pro-caspase-9 to create the apoptosome that finally activates the director caspase-3 [9, 10].

Circular RNAs (circRNAs) is a class of RNAs possessing many biological activities, such as sponging miRNA, affecting epithelial-to-mesenchymal transition (EMT)-related cellular functions, regulating RNA polymerase II transcription [11–14]. In addition, studies have shown that circRNAs could act as potential clinical biomarkers in complex disorders of the central nervous system [15], and alteration in the expressions of circRNAs was implicated in basal cell carcinoma [16], pathological hypertrophy and heart failure [17], laryngeal cancer [18].

In this study, we aimed to determine the damages in mouse lung tissue and the changes in circRNAs expression profiles upon exposure to radon, in order to explore their potential functions in radon-induced lung damage.

## Methods

### Animals and exposure conditions

All animal handling procedures were reviewed and approved by the Animal Care/User Ethical Committee of Soochow University, Suzhou City, P. R. China. 12 male BALB/c mice weighing approximately 15 g were obtained from Shanghai Laboratory Animal Center, and randomly divided into the experimental group and the control group with 6 mice per group. The mice in the experimental group were exposed to radon in a multifunctional radon chamber (Dong-hua University, China), at a concentration of 100,000 Bq/m^3^, 12 h/d, for up to cumulative doses of 60 working level months (WLM). The radon concentration in the exposure chamber was constantly maintained and continuously measured by scintillation counting. Food and water were available ad libitum during the exposure. Control mice were housed in a same room with a background concentration of radon at 20 Bq/m^3^ [19].

### Sample collection and microarray hybridization

After the last exposure, animals were sacrificed and the left lung tissue was used to extract the total RNA for circRNA analysis. Right lung was used for hematoxylin and eosin (H&E) staining, immunohistochemistry (IHC) analysis and Western blot assay.

Total RNA from each sample was quantified using the NanoDrop ND-1000. The sample preparation and microarray hybridization were performed based on the Arraystar’s standard protocols. Briefly, total RNA from each sample was amplified and transcribed into fluorescent circRNA using random primer according to Arraystar’s Super RNA Labeling protocol (Arraystar Inc.). The labeled circRNAs were hybridized onto the Arraystar Mouse circRNA Array (6x7K, Arraystar). After washing the slides, the arrays were scanned by the Axon GenePix 4000B microarray scanner. Scanned images were then imported into GenePix Pro 6.0 software (Axon) for grid alignment and data extraction. Quantile normalization and subsequent data processing were performed using the R software package. Differentially expressed circRNAs with statistical significance between the two groups were identified through Volcano Plot filtering. Differentially expressed circRNAs between two samples were identified through Fold Change filtering. Hierarchical Clustering was performed to show the distinguishable circRNA expression pattern among samples.

### Histological assessment and immunohistochemistry

H&E staining was conducted to examine histological pathology of lung tissue under a light microscope. Digital images were collected by a conventional light microscope in 5 visual fields/per section with 400× magnification under bright-field viewing. IHC stains were performed to examine the caspase-3 expression level in tissues from mice exposed to radon. The paraffin-embedded sections were stained with antibody against caspase-3. Tissue array slides were deparaffinized 3 times with Xylene, each for 5 min. The slides were rehydrated by a sequential treatment with 100% ethanol twice for 5 min each; 95% ethanol twice for 5 min each; and 80% ethanol twice for 5 min. Antigen retrieval was performed by microwaving in 0.01 M sodium citrate buffer (pH 6.0) at 96–100 °C for 15 min. Endogenous peroxidase activity was quenched with 3% hydrogen peroxide at room temperature for 25 min. After rinsing with PBS (pH 7.4) three times each for 5 min, the sections were then incubated with rabbit polyclonal antibody against caspase-3 (1:100 dilution, Abcam, USA) overnight at 4 °C. Following extensive washing with PBS, anti-rabbit secondary antibody (KPL, USA) was applied with 1:2000 dilutions for 1 h. The slides were then visualized using 3,3-diaminobenzidine (DAB) chromogen (LabVision Corp., Fremont, CA, USA) and counterstained with H&E. The sections were washed in PBS, dried, mounted in neutral gum, and observed at 400× magnification with a Nikon TE200 inverted microscope (Nikon Co, Japan). The sections for which the primary antibody was omitted were used as negative controls. 5 non-repeat high-power field images were randomly selected in which the brown particles were considered as positive areas.

### Western blot analysis

Total protein was extracted from lung tissues. The protein concentrations of individual samples were assessed using a standard bicinchoninic acid assay (Beyotime, Nantong, China). For each sample, 30 μg of protein was loaded on 10% SDS-PAGE gel (Bio-Rad, USA), transferred onto a polyvinylidene difluoride membrane and blocked with 5% skimmed milk and 0.1% Tris-buffered saline-Tween 20 (TBST) at room temperature for 1.5 h. The membranes were incubated overnight at 4 °C with caspase-3 antibody (1:1,000 dilution, Abcam, USA), mouse anti-GAPDH and mouse anti-β-Actin antibodies (1/8,000 dilution, Beyotime, Nantong, China). The membranes were then washed with TBST and incubated with anti-rabbit IgG horseradish peroxidase-conjugated secondary antibody (Cell Signaling Technology, Boston, MA). The protein expression was evaluated using chemiluminescence and exposure to Kodak film (Kodak, Rochester, NY).

### RNA Isolation and real-time PCR

Five highest expressing circRNAs were chosen to be examined in the mice of the 60 WLM group and the control group. The total RNA was extracted with TRIzol and reversely transcribed using SuperScriptTM III Reverse Transcriptase(Invitrogen, USA). The primers used in this assay are listed in Table 1. The following PCR conditions were used: 95 °C for 30 s, followed by 40 cycles of 95 °C for 10 s, 62 °C for 30 s. All RNA samples were analyzed in triplicate for each tested transcript and normalized to GAPDH. Expression fold changes were calculated using the 2^−ΔΔCt^ methods for quantitative analysis.

**Table 1:** Primer sequences

Gene	Sequence (5’-3’)
GAPDH	F:5’GTTGTCTCCTGCGACTTCA3’ R:5’GCCCCTCCTGTTATTATGG3’
mmu_circ_0001028	F:5’ TATTCTTCTGGGTGAGGATGGC3’ R:5’ TTGATCCGCTTTATGGCTACG3’
mmu_circ_0001311	F:5’ CCCCAGGATCTTCGTAGGTTA3’ R:5’ TCTTTCTTCCTTCAGCCACTTC3’
mmu_circ_0001154	F:5’ TCTGGAACATTGCGATTTGGA 3’ R:5’ TTGTTGACCGATGCCCACTC3’
mmu_circ_0001052	F:5’ CCGTACAGGGTTAAAGTGATAG3’ R:5’ TTTGATAAACATACGGTGGGT3’
mmu_circ_0001796	F:5’ GTCCGTACCCGATCAGTTGG 3’ R:5’ CGTTCTCAGACCTGCCTCCT 3’

### Statistical analysis

The SPSS 17.0 software was used for the statistical analysis. The data are presented as mean ± SEM. Group comparisons were evaluated by one-way ANOVA to determine statistical significance. Differences were considered significant when *P*<0.05.

## Results

### Alteration in histological pathology of mouse lung tissue

**Fig. 1:**
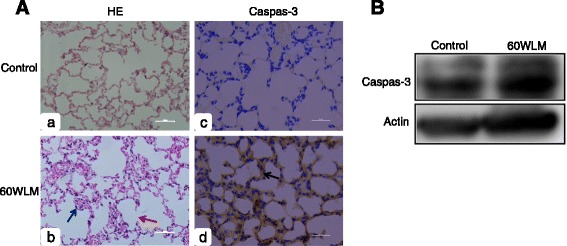
H&E and immunohistochemistry in mouse lung tissue exposed to radon. **A** Representative H&E and immunohistochemistry staining images of caspase-3 in normal mouse lung tissues and exposure samples. *Black arrow* indicates inflammatory cell in the alveolus interval, *red arrow* indicates alveolar septal fracture, *black arrow* indicates positive cells. **B** Western blot analysis of caspase-3 expression in mouse lung tissue

The H&E stainings of lung tissues from mice exposed to 60 WLM radon displayed inflammatory cells in the alveolus interval (Fig. 1A. b: black arrow) and some alveolar septal fracture (Fig. 1A. b: red arrow). Immunohistochemistry results showed that the brown represented positive cells. The expression of apoptotic activator protein caspase-3 was significantly increased in 60 WLM mice (Fig. 1A. d: black arrow). Western blot result showed that caspase-3 protein expression was also significantly increased compared with the control group (Fig. 1B).

### CircRNA expression profiles in control and 60WLM group

**Fig. 2:**
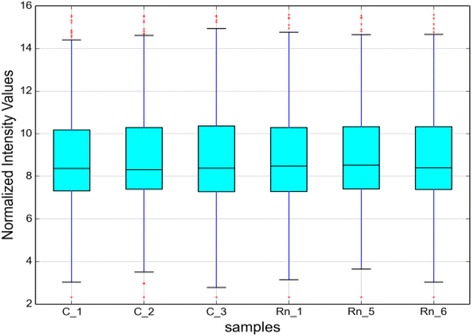
Box plot. X-axis: 6 tissue samples; Y-axis: normalized intensity values. All the 6 samples in the databases were normalized. The distribution of circRNAs was similar in all samples

Three mice from the control group and 60 WLM group, respectively, were chosen to assess the circRNA profiles using the circRNA microarray. A box view was used to compare the distribution of expression values for the samples after normalization. The distribution of log2 ratios was similar in all the tested samples as shown in Fig. 2.

**Fig. 3:**
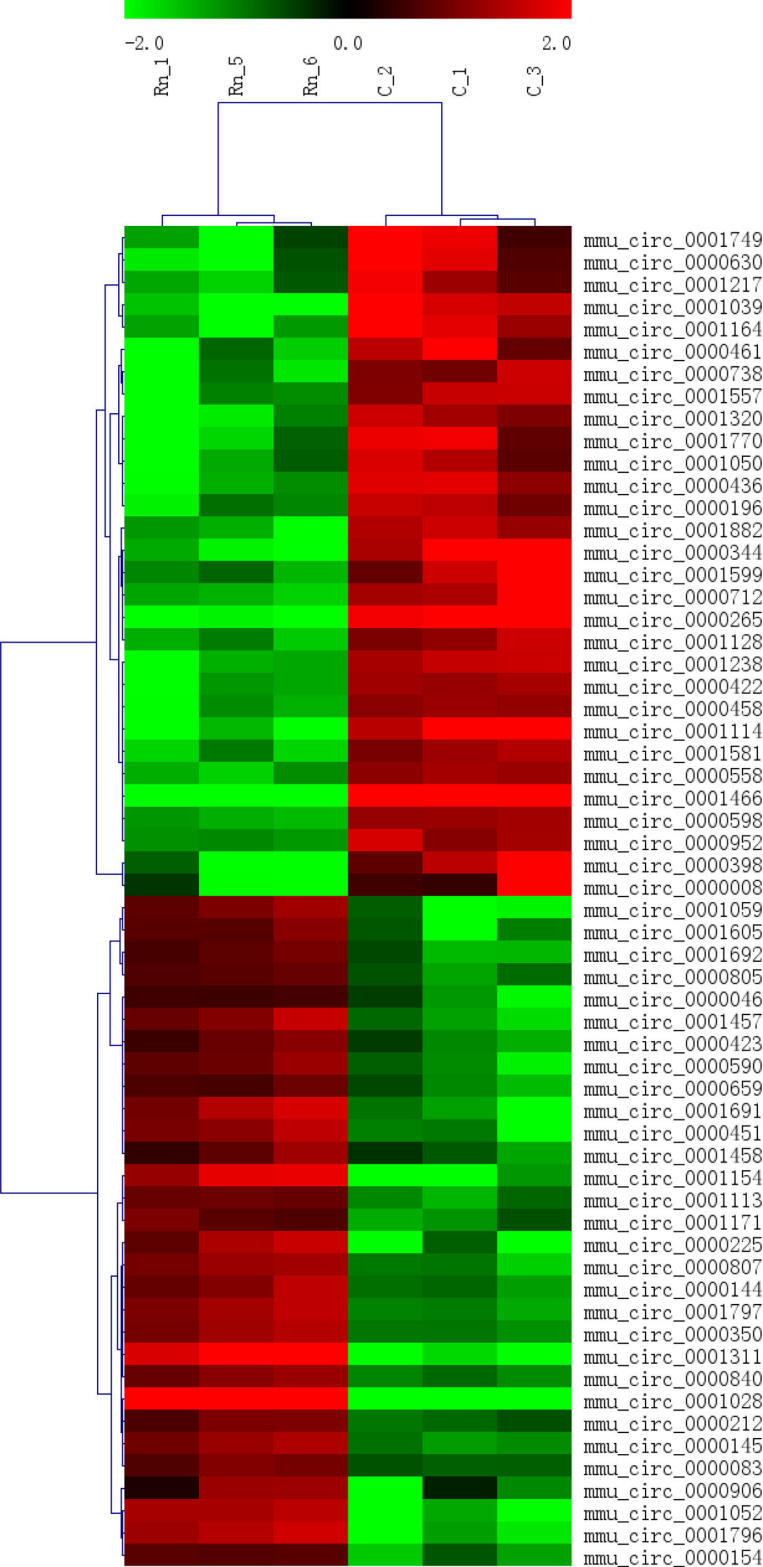
Top 30 up-regulated and down-regulated circRNAs were chose for analysis. Hierarchical cluster analysis diagram of the control and 60 WLM group (*n*=3) versus the sham-irradiated control (*n*=3). The hierarchical clustering is displayed as color saturation, which is directly proportional to the measured expression. The *color saturation* represents the log2 value of the normalized intensity of each sample (ranges from 7 to 11; *P* value cut-off 0.05). *Rows* indicate the individual probe set, and the columns represent the experimental sample

Hierarchical clustering is one of the simplest and widely used clustering techniques for gene expression data analysis. Cluster analysis arranges are based on their expression levels, which allows us to hypothesize on the relationships between two groups. The dendrogram shows the relationships of the expression levels between the two groups. In this study, hierarchical clustering was performed based on “All Targets Value - CircRNAs”. The result showed that a distinguishable mRNA expression profiling between the control and radon-exposed groups as shown in Fig. 3.

**Fig. 4:**
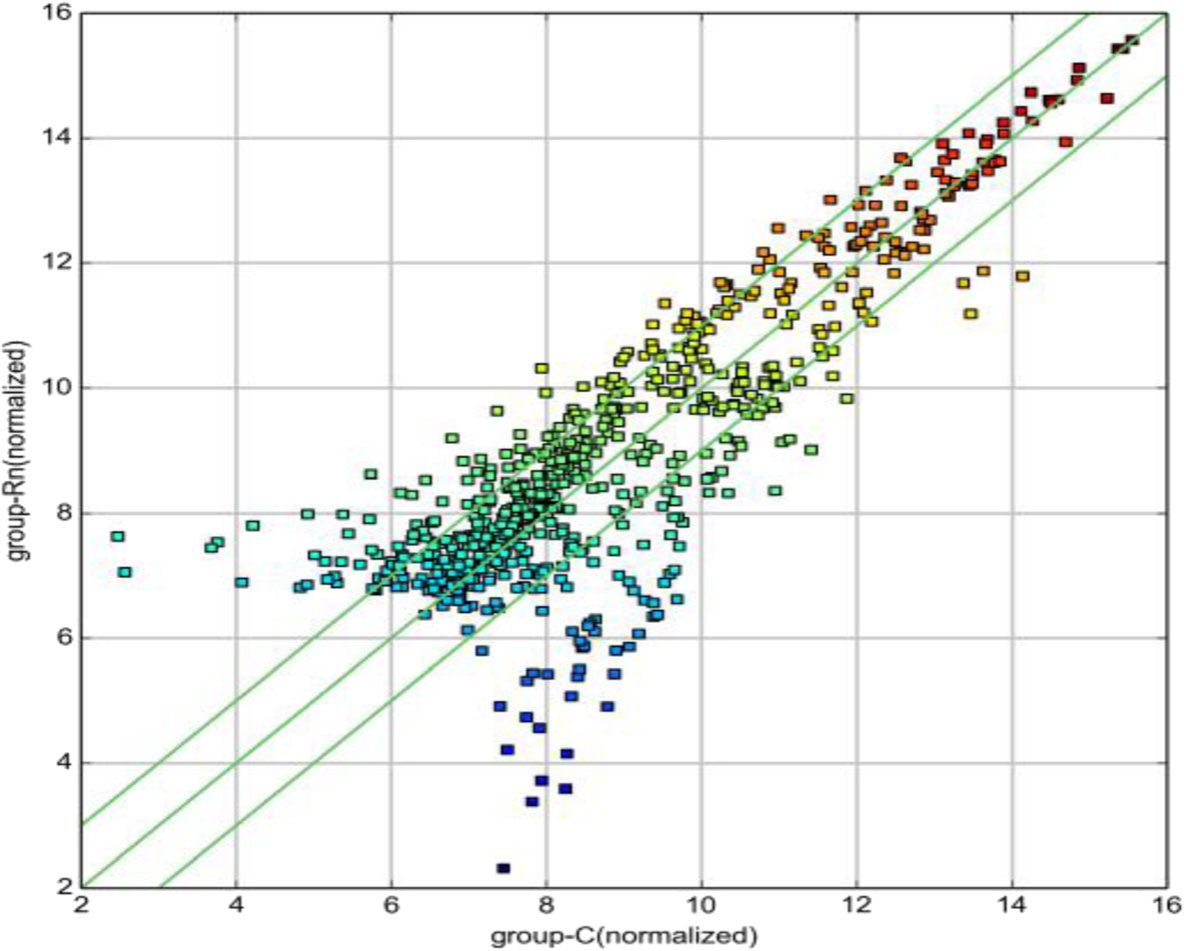
Scatter plot. X-axis: adjacent non-tumor tissues (normalized), Yaxis: LSCC tissues (normalized). The *green lines* represent fold change. The circRNAs above the top *green line* and below the *bottom green line* indicate more than 2 fold change in circRNA expressions between the two groups of samples

The Scatter plot was used to assess the variations in circRNA expressions between the lung tissues of the control and exposure groups. The values of X and Y axes in the Scatter plot are the normalized signal values of the samples (log2 scaled) or the averaged normalized signal values of groups of samples (log2 scaled). The green lines are fold change lines. The circRNAs above the top green line and below the green line on the bottom indicated more than 2 fold change in circRNAs between the two groups as shown in Fig. 4.

**Fig. 5:**
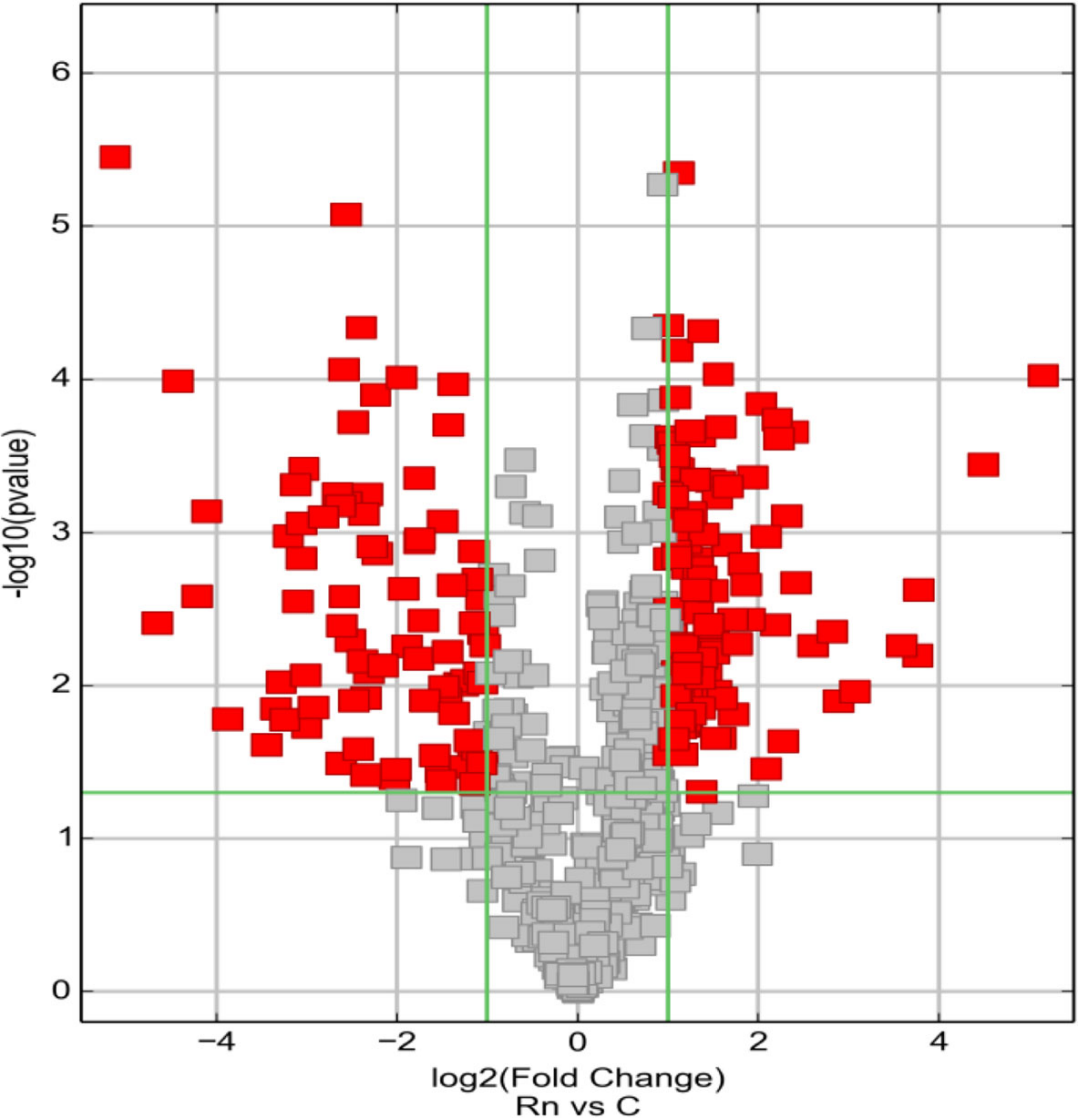
Volcano plot. X-axis: log2 (fold change); Y-axis: −log10 (*P*-value). The vertical green lines represent 2 fold up-regulation (*left*) and down-regulation (*right*), and the horizontal *green line* represents a *p*-value of 0.05. The *red points* in the plot represent circRNAs expressed differentially with statistical significance

The Volcano plot was performed to visualize the differential expressions between the control and exposed groups. The significant differences (fold change >2.0, *P* < 0.05) between the two different conditions are illustrated in the Volcano plot, and the red points in the plot represents the differentially expressed circRNAs with statistical significance as shown in Fig. 5.

### CircRNA expression profile and function prediction

Microarray screening revealed 107 up-regulated and 83 down-regulated circRNAs, among which the top 30 circRNAs with the most fold changes were chosen for further analysis, with 5 microRNA binding sites were listed for each circRNA (Tables 2 and 3).

**Table 2:** Fold change of top 30 circRNAs up-regulation

CircBase ID	Fold Change	best_transcript	GeneSymbol	miRNA Binding Sites 1	miRNA Binding Sites 2	miRNA Binding Sites3	miRNA Binding Sites4	miRNA Binding Sites5
mmu_circ_0001028	35.63598	NM_016866	Stk39	mmu-miR-703	mmu-miR-879-3p	mmu-miR-3058-5p	mmu-miR-693-3p	mmu-miR-7015-5p
mmu_circ_0001311	22.46768	NM_001003909	Ankib1	mmu-miR-103-3p	mmu-miR-107-3p	mmu-miR-7092-3p	mmu-miR-712-5p	mmu-miR-6935-3p
mmu_circ_0001154	13.69381	NM_133869	Cept1	mmu-miR-6946-3p	mmu-miR-22-5p	mmu-miR-1955-5p	mmu-miR-298-5p	mmu-miR-500-5p
mmu_circ_0001052	13.58701	NM_001145824	Hipk3	mmu-miR-7222-5p	mmu-miR-1929-5p	mmu-miR-338-3p	mmu-miR-6922-3p	mmu-miR-143-5p
mmu_circ_0001796	12.02444	NM_172536	Zfp609	mmu-miR-7009-3p	mmu-miR-3971	mmu-miR-6974-3p	mmu-miR-6975-3p	mmu-miR-344 g-3p
mmu_circ_0001691	8.370497	NM_030113	Arhgap10	mmu-miR-6898-3p	mmu-miR-7221-5p	mmu-miR-201-3p	mmu-miR-210-5p	mmu-miR-326-3p
mmu_circ_0001059	7.40114	NM_029617	Casc5	mmu-miR-6344	mmu-miR-7035-5p	mmu-miR-28a-5p	mmu-miR-7233-3p	mmu-miR-28c
mmu_circ_0000225	7.036664			mmu-miR-8092	mmu-miR-875-3p	mmu-miR-7679-3p	mmu-miR-532-5p	mmu-miR-6481
mmu_circ_0000451	6.093728	NM_001123386	Cdyl	mmu-miR-7083-5p	mmu-miR-6902-5p	mmu-miR-185-3p	mmu-miR-3085-3p	mmu-miR-3064-5p
mmu_circ_0001457	5.334011	NM_013791	Mkln1	mmu-miR-30d-3p	mmu-miR-3071-3p	mmu-miR-7214-5p	mmu-miR-5615-5p	mmu-miR-7b-5p
mmu_circ_0001797	5.213906	NM_172536	Zfp609	mmu-miR-7009-3p	mmu-miR-3971	mmu-miR-6974-3p	mmu-miR-6975-3p	mmu-miR-6961-3p
mmu_circ_0000807	4.974568	NM_001025572	Ankrd12	mmu-miR-7092-3p	mmu-miR-683	mmu-miR-6944-3p	mmu-miR-7684-3p	mmu-miR-6899-3p
mmu_circ_0001605	4.840995	NM_175388	Rnf169	mmu-miR-3095-5p	mmu-miR-693-3p	mmu-miR-207	mmu-miR-6344	mmu-miR-7038-3p
mmu_circ_0000145	4.680084	NM_011682	Utrn	mmu-miR-6958-3p	mmu-miR-1968-5p	mmu-miR-22-3p	mmu-miR-19b-2-5p	mmu-miR-450b-5p
mmu_circ_0000350	4.620216	NM_001162465	Dtnb	mmu-miR-3066-5p	mmu-miR-6938-3p	mmu-miR-6929-5p	mmu-miR-103-1-5p	mmu-miR-103-2-5p
mmu_circ_0000590	4.569337	NM_018745	Azin1	mmu-miR-216a-5p	mmu-miR-7657-3p	mmu-miR-489-5p	mmu-miR-203-5p	mmu-miR-5625-3p
mmu_circ_0000144	4.247965	NM_011682	Utrn	mmu-miR-6958-3p	mmu-miR-1968-5p	mmu-miR-22-3p	mmu-miR-328-3p	mmu-miR-19b-2-5p
mmu_circ_0000906	4.241155	ENSMUST00000182122	Zfp236	mmu-miR-1930-3p	mmu-miR-143-5p	mmu-miR-669d-5p	mmu-miR-1187	mmu-miR-466i-5p
mmu_circ_0000840	4.08919	NM_001039692	Arhgap12	mmu-miR-30c-1-3p	mmu-miR-450a-5p	mmu-miR-30c-2-3p	mmu-miR-592-5p	mmu-miR-320-5p
mmu_circ_0001113	3.831634	NM_173182	Fndc3b	mmu-miR-93-3p	mmu-miR-7231-3p	mmu-miR-412-3p	mmu-miR-6998-3p	mmu-miR-298-5p
mmu_circ_0001692	3.745827	NM_030113	Arhgap10	mmu-miR-6898-3p	mmu-miR-201-3p	mmu-miR-326-3p	mmu-miR-7011-3p	mmu-miR-7b-5p
mmu_circ_0000154	3.65036	NM_018747	Akap7	mmu-miR-1903	mmu-miR-135b-5p	mmu-miR-135a-5p	mmu-miR-106a-5p	mmu-miR-93-5p
mmu_circ_0001171	3.562626	NM_146141	Ppa2	mmu-miR-7092-3p	mmu-miR-6349	mmu-miR-206-3p	mmu-miR-6382	mmu-miR-1a-3p
mmu_circ_0000423	3.392009	NM_011625	Ppp1r13b	mmu-miR-7649-3p	mmu-miR-6946-3p	mmu-miR-7083-3p	mmu-miR-3473c	mmu-miR-1903
mmu_circ_0000046	3.298026	NM_009952	Creb1	mmu-miR-5623-5p	mmu-miR-6911-5p	mmu-miR-7688-5p	mmu-miR-7242-3p	mmu-miR-216a-3p
mmu_circ_0000659	3.281563	NM_029582	Txndc11	mmu-miR-21a-3p	mmu-miR-5110	mmu-miR-7038-5p	mmu-miR-331-3p	mmu-miR-3064-3p
mmu_circ_0000212	3.168443	NM_001037846	Cnot2	mmu-miR-6902-5p	mmu-miR-6914-3p	mmu-miR-7052-5p	mmu-miR-7214-3p	mmu-miR-6972-5p
mmu_circ_0000805	3.13632	NM_013933	Vapa	mmu-miR-1298-5p	mmu-miR-672-3p	mmu-miR-19b-1-5p	mmu-miR-7212-3p	mmu-miR-19b-2-5p
mmu_circ_0001458	3.029251	NM_013791	Mkln1	mmu-miR-296-5p	mmu-miR-8103	mmu-miR-135a-5p	mmu-miR-135b-5p	mmu-miR-30d-3p
mmu_circ_0000083	2.999915	NM_001166501	Dennd1b	mmu-miR-145a-5p	mmu-miR-145b	mmu-miR-122-5p	mmu-miR-103-3p	mmu-miR-107-3p

**Table 3:** Fold change of top 30 circRNAs down-regulation

CircBase ID	Fold Change	best_transcript	GeneSymbol	miRNA Binding Sites 1	miRNA Binding Sites 2	miRNA Binding Sites3	miRNA Binding Sites4	miRNA Binding Sites5
mmu_circ_0001466	34.8883803	NM_194061	D630045J12Rik	mmu-miR-6390	mmu-miR-468-3p	mmu-miR-7232-3p	mmu-miR-29b-2-5p	mmu-miR-146a-3p
mmu_circ_0001039	25.2731871	NM_016965	Nckap1	mmu-miR-7065-3p	mmu-miR-5619-5p	mmu-miR-7010-3p	mmu-miR-143-5p	mmu-miR-6344
mmu_circ_0000265	21.5734915	NM_173753	Fnip1	mmu-miR-221-5p	mmu-miR-145a-3p	mmu-miR-146a-3p	mmu-miR-504-5p	mmu-miR-3113-5p
mmu_circ_0000344	18.5911228	NM_029878	Tbcd	mmu-miR-3474	mmu-miR-7239-3p	mmu-miR-6945-5p	mmu-miR-1982-5p	mmu-miR-204-3p
mmu_circ_0001114	17.2864639	NM_001163007	Tnik	mmu-miR-384-5p	mmu-miR-199a-3p	mmu-miR-199b-3p	mmu-miR-543-3p	mmu-miR-30e-5p
mmu_circ_0000398	14.6857941	NM_001167920	Slc8a3	mmu-miR-8113	mmu-miR-6900-5p	mmu-miR-6957-5p	mmu-miR-3552	mmu-miR-7050-5p
mmu_circ_0000738	10.8968513	NM_027060	Btbd9	mmu-miR-15b-5p	mmu-miR-15a-5p	mmu-miR-322-5p	mmu-miR-16-5p	mmu-miR-195b
mmu_circ_0000461	10.1374001	NM_011078	Phf2	mmu-miR-29b-2-5p	mmu-miR-29b-1-5p	mmu-miR-191-3p	mmu-miR-337-3p	mmu-miR-673-3p
mmu_circ_0001770	9.7481856	NM_008775	Pafah1b2	mmu-miR-1224-3p	mmu-miR-683	mmu-miR-6935-3p	mmu-miR-3092-5p	mmu-miR-6342
mmu_circ_0001557	9.492165	NM_016682	Uba2	mmu-miR-7649-3p	mmu-miR-6399	mmu-miR-7226-5p	mmu-miR-380-5p	mmu-miR-3090-5p
mmu_circ_0001164	9.234634	TCONS_00021080	XLOC_016164	mmu-miR-7062-5p	mmu-miR-7116-3p	mmu-miR-106a-3p	mmu-miR-691	mmu-miR-7093-3p
mmu_circ_0001238	8.7123832	NM_001080926	Lrp8	mmu-miR-7032-5p	mmu-miR-7094b-2-5p	mmu-miR-6990-5p	mmu-miR-674-5p	mmu-miR-1956
mmu_circ_0001320	8.5860481	NM_010305	Gnai1	mmu-miR-7011-3p	mmu-miR-6360	mmu-miR-1291	mmu-miR-324-3p	mmu-miR-3105-3p
mmu_circ_0000436	8.3430278	NM_172120	Vps41	mmu-miR-7b-5p	mmu-miR-670-3p	mmu-miR-7034-3p	mmu-miR-7117-3p	mmu-miR-7093-3p
mmu_circ_0001882	8.3381778	NM_001160403	Il1rapl1	mmu-miR-337-3p	mmu-miR-7018-5p	mmu-miR-8095	mmu-miR-195b	mmu-miR-6975-3p
mmu_circ_0000712	8.1893468	NM_139145	Hlcs	mmu-miR-5133	mmu-miR-6938-5p	mmu-miR-7118-5p	mmu-miR-6911-5p	mmu-miR-7672-5p
mmu_circ_0000630	8.0822088	NM_172610	Mpped1	mmu-miR-7037-5p	mmu-miR-5120	mmu-miR-7062-5p	mmu-miR-5132-5p	mmu-miR-6897-5p
mmu_circ_0001749	8.0148488	NM_145610	Ppan	mmu-miR-6954-5p	mmu-miR-6938-3p	mmu-miR-6914-5p	mmu-miR-486b-3p	mmu-miR-486a-3p
mmu_circ_0001050	7.5953109	NM_010163	Ext2	mmu-miR-7019-5p	mmu-miR-30b-3p	mmu-miR-3547-5p	mmu-miR-6982-5p	mmu-miR-6930-5p
mmu_circ_0000422	7.0486583	NM_021516	Mark3	mmu-miR-466f	mmu-miR-1187	mmu-miR-574-5p	mmu-miR-709	mmu-miR-297a-5p
mmu_circ_0000458	6.3303882	NM_019930	Ranbp9	mmu-miR-3109-5p	mmu-miR-145b	mmu-miR-490-3p	mmu-miR-7007-5p	mmu-miR-5124a
mmu_circ_0001581	6.1968327	NR_028143	Lrrc28	mmu-miR-3087-5p	mmu-miR-1231-5p	mmu-miR-6998-5p	mmu-miR-1912-5p	mmu-miR-34a-5p
mmu_circ_0000008	6.143468	NM_026493	Cspp1	mmu-miR-1903	mmu-miR-3154	mmu-miR-6340	mmu-miR-203-5p	mmu-miR-6946-3p
mmu_circ_0001599	6.1137112	NM_011035	Pak1	mmu-miR-1903	mmu-miR-6516-5p	mmu-miR-107-5p	mmu-miR-181d-5p	mmu-miR-6946-3p
mmu_circ_0000558	6.0158681	NM_001164503	Akap11	mmu-miR-452-5p	mmu-miR-6925-5p	mmu-miR-6999-5p	mmu-miR-6976-5p	mmu-miR-7241-5p
mmu_circ_0000196	6.0061817	NM_001277188	Ano4	mmu-miR-691	mmu-miR-5622-5p	mmu-miR-302a-3p	mmu-miR-5620-3p	mmu-miR-20b-5p
mmu_circ_0000598	5.9125661	NM_001113554	Nudcd1	mmu-miR-494-5p	mmu-miR-6946-3p	mmu-miR-7054-5p	mmu-miR-6935-3p	mmu-miR-376b-5p
mmu_circ_0001128	5.9008556	NM_001198766	Postn	mmu-miR-29b-2-5p	mmu-miR-29b-1-5p	mmu-miR-23a-5p	mmu-miR-344d-2-5p	
mmu_circ_0001217	5.7224481	NM_001163732	Frmd3	mmu-miR-7024-3p	mmu-miR-6939-5p	mmu-miR-7663-5p	mmu-miR-1967	mmu-miR-450a-1-3p
mmu_circ_0000952	5.6172869	NM_145501	Pi4k2a	mmu-miR-1907	mmu-miR-133c	mmu-miR-133a-3p	mmu-miR-133b-3p	mmu-miR-6989-3p

### Identification of circRNAs

**Fig. 6:**
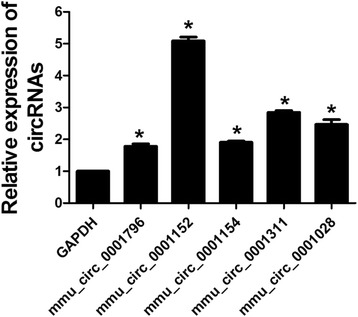
The expressions of 5 circRNAs were examined in mice of 60 WLM group. Real-time PCR was used to determine the expressions of the 5 highest circRNAs in the 60 WLM group. * *P*<0.05 vs GAPDH

Five circRNAs with the highest expressions were selected to validate the data from microarray in mice exposed to 60 WLM of radon as shown in Fig. 6. The result confirmed the consistency between data from microarray and real-time PCR.

## Discussion

Radon (^222^Rn) gas is usually released from rocks and soils, and rapidly disperses in the atmosphere. When radon is inhaled into the human respiratory system, the entire body could be subjected to internal exposure. Many reports showed that radon causes cancers, including lung cancer and childhood leukemia [1–4]. There have been strong evidences showing a significantly increased risk of lung cancer followed by long-term low radon exposure among Wismut miners [1]. Genome damage, such as genome instability, was found in peripheral blood lymphocytes of lung cancer patients living in a region with high air pollution and increased background radon levels [5, 20]. Moreover, IL-6 promoter variant was associated with lung cancer in uranium miners [21]. Reports show that caspase-3 is involved in cell apoptosis [22, 23]. Our results showed that caspas-3 expression was significantly increased in radon-exposed group, suggesting that the lung tissue was severely injured when exposed to radon.

CircRNA is an abundant, developmentally regulated, tissue and cell-type specific, stable transcriptional product, and is evolutionarily conserved across the eukaryotic tree of life [24, 25]. Studies showed that interactions between circRNAs and miRNAs indicate that circRNAs are potentially associated with many disease, such as gastric cancer [26], colorectal cancer [27] and breat cancer [28]. Since circRNAs are involved in many kinds of diseases and provide new insights for exploring the disease pathological mechanisms. In this study, we have identified 107 up-regulated and 83 down-regulated circRNAs. 5 miRNAs of each circRNA was successfully predicted. These circRNAs and miRNAs could play important and synergistic roles in radon-induced lung damage, even in lung cancer. Further studies need to be conducted to illustrate the functional mechanisms of circRNAs in radon-induced lung damages.

Recent evidence suggests that circRNAs play a crucial role in fine-tuning the level of miRNA-mediated regulation of gene expression by sequestering miRNAs. MiRNA response elements (MREs) were found in some circRNAs, which could sponge miRNAs, such as miR-7, miR-17, and miR-214 [29]. In addition, circRNA could affect miRNA activities and increase the complexity of RNA regulatory networks hence playing a role in gene expression [30]. Evidences indicated that circRNAs play important roles in tumorigenesis. For instance, ciRS-7 was found to inhibit cancer via miR-7 by down-regulating some critical oncogenes [31]. Hsa_circ_002059 expression in gastric cancer was involved in TNM stage, gender and age [32]. Moreover, hsa_circRNA_100855 and hsa_circRNA_104912 were involved in lymph node metastasis, poor differentiated or advanced clinical stages in laryngeal cancer [18]. In summary, these results showed that circRNAs may serve as potential biomarkers in certain diseases such as cancer. However, few circRNA/miRNA interactions have been experimentally validated. Therefore, the functional effect of their interaction will be the focus of our future research.

## Conclusion

In summary, our results suggest that mouse lung tissue was severely injured when exposed to radon through and a series of differentially expressed circRNAs were found. It will hopefully to further research the biological effects induced by radon.

## Abbreviations

circRNA: Circular RNA
EMT: Epithelial-to-mesenchymal transition
IHC: Immunohistochemistry
TBST: Tris-buffered saline-Tween
WLM: Working level months
MREs: MiRNA response elements
